# Safer Prescribing in Behavioural and Psychological Symptoms of Dementia (BPSD): Reducing Anticholinergic Burden

**DOI:** 10.1192/bjo.2025.10402

**Published:** 2025-06-20

**Authors:** Jessica May, Nurul Ain Mohd Nizam, Elisabeth Henrika Bonor, Louisa Marchant-Rutherford, Scott Cherry

**Affiliations:** 1 Sussex Partnership NHS Foundation Trust, Brighton, United Kingdom; 2 University Hospitals Sussex NHS Foundation Trust, Brighton, United Kingdom

## Abstract

**Aims:** NICE guidelines (NG97) emphasize the importance of assessing the anticholinergic burden of medications in older adults. Anticholinergic side effects of medications can worsen constipation, urinary retention, sedation and confusion, exacerbating cognition, falls and BPSD risks.

Medichec, a free online tool, measures medication effects on cognition and evaluates the cumulative impact. The tool provides an Anticholinergic Effect on Cognition (AEC) score for each medication, ranging from 0–3. The greater the score, the greater the need to evaluate its benefits versus risks.

This project aimed to evaluate the effectiveness of integrating the AEC score into multidisciplinary management of dementia inpatients.

**Methods:** In a 10-bed specialist dementia ward, AEC score was calculated weekly over four weeks for each patient using Medichec. This included 15 inpatients over 4 weeks. Medications contributing to AEC score were recorded and reviewed during the weekly ward multidisciplinary meetings. Individual plans were then made to reduce, hold, or stop medication where appropriate.

**Results:** Total AEC scores were between 1–7 for each patient, scoring primarily for psychotropic medications. The weekly percentage of patients with total AEC scores over 3 on the ward ranged between 10–37.5%. The overall trend in percentage of patients with a score over 3 showed a reduction from 37.5% at baseline to 20% at week 4.

There were 10 occasions where patients had an AEC score of 3+; on 90% of these occasions there was a documented multidisciplinary care plan and these 10 occasions represented 5 individual patients. Of these 5 patients, medications were adjusted for 2 patients, continued for 2 patients and not discussed for 1 patient.

The mean AEC score varied between 1.5–2.13; there was no reduction from baseline (1.89) to week 4 (1.9) however the average in week 4 was skewed by an outlying individual result of 7.

**Conclusion:** The weekly data collected in this audit supports a culture of avoiding medications with high anticholinergic burden for physical health reasons e.g. antimuscarinics for incontinence. Additionally, medications for insomnia, like 'Z-drugs', are not routinely prescribed. This helps lower the risks of side effects and aligns with NICE (NG97) guidelines for managing medications which can adversely affect cognition.

Medications with an individual anticholinergic score of 2+ or patients with a total score of 3+ on Medichec should trigger a review as this can have a clinically significant impact on individual patients.

